# Comparison of myopia control efficacy between single vision and highly aspherical Lenslet spectacle lenses in children with low myopia: a 1-year retrospective study

**DOI:** 10.3389/fmed.2026.1770828

**Published:** 2026-04-17

**Authors:** Shuang Wang, Keke Huang, Xingyu He, Zhanfeng Wang

**Affiliations:** Ophthalmology Department, Chengdu Third People’s Hospital, Affiliated Hospital of Southwest Jiaotong University, Chengdu, Sichuan, China

**Keywords:** age, highly aspherical Lenslets lenses, myopia, single vision lenses, spherical equivalent refraction

## Abstract

**Objective:**

This study aimed to evaluate the effectiveness of Single Vision Lenses (SVL) and Highly Aspherical Lenslets (HAL) lenses in the management of low myopia.

**Methods:**

This retrospective study included school-age children (aged 6–12 years) diagnosed with low myopia who were treated at Chengdu Third People’s Hospital from January 2022 to August 2025. Patients were divided into two groups based on their myopia control intervention: the SVL group and the HAL group. Subgroup analyses were subsequently performed within both groups according to age. The progression in mean spherical equivalent refraction (SER) and axial length (AL) was compared among the different groups and subgroups.

**Results:**

A total of 157 children (87 males [55.41%] and 70 females [44.59%]) with myopia (right eye only, 157 eyes), aged 6–12 years (mean age, 8.94 ± 1.39 years), were included. The SVL group comprised 49 cases (49 eyes) with an average progression in SER (ΔSER) of −0.70 ± 0.45 D and an average axial length increase (ΔAL) of 0.31 ± 0.19 mm. The HAL group comprised 108 cases (108 eyes) with an average ΔSER of −0.18 ± 0.38 D and an average ΔAL of 0.13 ± 0.16 mm. Within the HAL subgroups, the median ΔAL in the 7-year group was significantly higher than in the 8-year (*p* = 0.03), 9-year (*p* = 0.009), and 10-year (*p* = 0.003) groups. Within the SVL subgroups, the 8-year group had significantly higher ΔAL than the 9-, 10-, and 11-year groups (*p* < 0.05).

**Conclusion:**

Single-vision lenses provide only minimal control of myopia progression, whereas specially designed lenses such as HAL lenses effectively slow myopia development—especially by curbing axial elongation. Age is also a critical factor: even with HAL lenses, axial growth remains pronounced in children under 8 years and is more difficult to control. For those who develop myopia at a younger age, combined pharmacological interventions may offer a better strategy than optical correction alone.

## 1 Introduction

Myopia is a condition where, in a relaxed state, parallel light rays focus in front of the retina after passing through the eye’s refractive system. According to China’s National Health Commission, the overall myopia rate among children and adolescents in China had reached 52.7%, with rates of 80.5% among high school students, 71.1% among junior high school students, and 35.6% among primary school students (National Health Commission of the PRC, 2026). Furthermore, myopia has become a global public health issue not limited to only China: approximately 50% of the populations of several industrialized countries have myopia (1), and it is estimated that by 2050, nearly 4.8 billion people worldwide will be affected by myopia (2). Myopia not only leads to low vision but is also a risk factor for several eye diseases, such as cataract, glaucoma, retinal detachment, and macular degeneration (3). It is also one of the leading causes of blindness globally (4).

In East Asia, a rapid increase in the prevalence of myopia among school-age children aged between 6 and 12 years has been reported: a study in Hong Kong showed that the incidence rate of myopia among 6-year-olds was 18.3%, and by the age of 12 years, the incidence rate rose to approximately 61.5% (5); another study in Taiwan reported that the incidence rate of myopia among 7-year-olds is currently 25.41%, and by the age of 12, this increases to approximately 76.67% (6). Several children develop myopia while in primary school, and the degree of myopia increases rapidly. Therefore, it is essential to control myopia in school-aged children in primary schools.

Since myopia is incurable, its prevention and control have become the current priority. There are three widely used and effective control measures: corneal contact lenses (multifocal soft contact lenses, orthokeratology lenses), spectacle lenses (highly aspherical Lenslets (HAL) spectacle lenses, Defocus Incorporated Multiple Segments (DIMS) lenses, and progressive lenses), and administration of low concentrations of atropine (7). These control measures are all effective but the spectacle lenses are the most common and are considered a non-invasive option with a high safety profile. There are many comparative studies on HAL lenses and single vision lenses, but there is little research on the age influence of HAL lenses (3, 7). This article aims to compare the different effectiveness of HAL lenses and single vision lenses (SVL) in Chinese children aged between 6 and 12 years in low myopia and the difference of HAL between different ages.

## 2 Materials and methods

### 2.1 Study design

In this retrospective study, we collected the data of school-age children (aged, 6–12 years) with myopic who were treated at the Chengdu Third People’s Hospital in Chengdu, China, from January 2022 to August 2025. The patients’ data that were collected included their age, sex, spherical equivalent refraction (SER), axial length of the eye, the use of single vision lenses, the use of highly aspherical Lenslets (HAL) spectacle lenses (produced by Essilor, France). The Patients were then categorized based on their myopia control intervention: the SVL group and the HAL group. Subsequently, the HAL group was further divided into age-based subgroups for analysis. Each patient had regular follow-ups every 6 months, and data were recorded for a period of 1 year. To account for the correlation between two eyes of the same subject, only data from the right eye were included in the final statistical analysis. All consecutive eligible patients treated during the study period were enrolled, and no *a priori* sample size calculation was performed. The ratio between the HAL and SVL groups was not predetermined but reflected the actual distribution of myopia control interventions among the study population.

### 2.2 Inclusion and exclusion criteria

The inclusion criteria were as follows: (1) initial age of the child between 6 and 12 years; and (2) Myopia was confirmed by cycloplegic refraction, with spherical equivalent refraction ranging from −0.50 D to −3.00 D. The exclusion criteria were as follows: (1) the presence of any systemic diseases affecting vision; (2) other diagnosed ophthalmic diseases, including congenital cataracts, retinal detachment, retinoblastoma, keratoconus, etc.; (3) exophoria of more than 3 prism diopters, intermittent exotropia, and concomitant exotropia, as well as other forms of strabismus; (4) refractive disparity; and (5) early-onset myopia occurring within 6 years of age.

### 2.3 Examination items and criteria

1. *Cycloplegic Refraction*: All children were administered one drop per eye of topical tropicamide eye drops (0.5%, manufactured by Shandong Bausch and Lomb FruiTech Pharmaceuticals Co. Ltd., China), and this was repeated six times at 5-min intervals. After cycloplegia, retinoscopy was performed, followed by subjective refraction. All refractives were recorded using the spherical equivalent refraction (SER).
2. *Axial Length Measurement*: Axial length measurements were conducted using the IOL-MASTER700 (produced by Carl Zeiss Meditec, Germany), with five repeated measurements taken per eye, and the average value was recorded for analysis. The data collected were recorded to two decimal places.
3. *Spectac*les: All eligible children with myopia exceeding −0.50 D were prescribed either single vision lenses (SVL) or highly aspherical Lenslets (HAL) spectacle lenses. Full correction was provided based on cycloplegic refraction, and under-correction was not permitted during the follow-up period. Re-prescription or lens replacement was performed when the spherical equivalent refraction changed by ≥0.50 D or when best-corrected visual acuity decreased by ≥2 lines, as assessed at the 6-month follow-up visits.

### 2.4 Statistical analysis

All analyses were performed with SPSS 19.0 (IBM SPSS Statistics 19.0). For continuous variables, normality (Shapiro–Wilk test) and homogeneity of variances (Levene test) were examined first. Between-group comparisons: if data were normally distributed and variances were equal, an independent-samples *t*-test was used; otherwise, the Mann–Whitney *U* test was applied. Categorical variables (e.g., sex) were analyzed with Pearson’s *χ*^2^ test or Fisher’s exact test when expected counts were <5.

Within the HAL and SVL group, age-subgroup comparisons (7-, 8-, 9-, 10-, 11-, and 12-year groups) were conducted as independent samples.

- Normal and equal variance: one-way ANOVA followed by LSD or Tukey post-hoc tests when the overall effect was significant.
- Normal but unequal variance: Welch ANOVA with Games–Howell post-hoc tests.
- Non-normal: Kruskal–Wallis *H* test; when significant, pairwise comparisons were performed using the Dwass–Steel–Critchlow–Fligner (DSCF) method.

A two-tailed *p*-value < 0.05 was considered statistically significant.

To avoid inter-eye correlation, only data from the right eye were included in the primary analysis. This approach is commonly adopted in ophthalmic studies to eliminate bias arising from within-subject interdependence. To confirm the robustness of the findings, we additionally performed a sensitivity analysis using data from the left eye alone. The results were consistent with those obtained from the right eye (data not shown), indicating that the conclusions were not affected by eye selection.

## 3 Results

### 3.1 Participants general characteristics

A total of 157 children (87 male children (55.41%) and 70 female children (44.59%)) with myopia (choosing the right eye including 157 eyes), aged between 6 and 12 years (mean age, 8.94 ± 1.39 years) were included in this study. The distribution across the groups was as follows: the SVL group had 49 cases involving 49 eyes, the HAL group had 108 cases involving 108 eyes. In the SVL group, there were 27 male and 22 female children (mean age, 9.12 ± 1.45 years) with an average progression in SER (ΔSE) of −0.70 ± 0.45 D and an average axial length increase (ΔAL) of 0.31 ± 0.19 mm. In the HAL group, there were 60 male and 48 female children (mean age, 8.86 ± 1.37 years) with an average progression in SER of −0.18 ± 0.38D and an average axial length increase of 0.13 ± 0.16 mm. The basic characteristics of the two groups are listed in Table 1.

**Table 1:** Baseline and 1-year follow-up data of the SVL group and HAL group.

Variable	SVL group	HAL group	*p*-value
	*n* = 49	*n* = 108	
Eye number	49	108	
Age (years)	9.12 ± 1.45	8.86 ± 1.37	0.244
Sex, male/female	27/22	60/48	0.93
Initial spherical equivalent refraction, SER1 (D)	−1.38 ± 0.80	−1.54 ± 0.79	0.154
Spherical equivalent refraction progression (ΔSER) (D)	−0.70 ± 0.45	−0.18 ± 0.38	<0.001
Initial axial length (AL1) (mm)	24.13 ± 0.87	24.18 ± 0.61	0.591
Axial length elongations (ΔAL) (mm)	0.31 ± 0.19	0.13 ± 0.16	<0.001

Within the HAL group, participants were stratified by age (6–12 years) into seven subgroups (Table 2): 6 years (*n* = 2), 7 years (*n* = 15), 8 years (*n* = 27), 9 years (*n* = 36), 10 years (*n* = 13), 11 years (*n* = 10), and 12 years (*n* = 5). Within the SVL group, participants were stratified by age (6–12 years) into seven subgroups (Table 3): 6 years (*n* = 1), 7 years (*n* = 6), 8 years (*n* = 9), 9 years (*n* = 15), 10 years (*n* = 9), 11 years (*n* = 6), and 12 years (*n* = 3).

**Table 2:** Baseline and 1-year follow-up data by age within the HAL group.

Variable	Age	Age	Age	Age	Age	Age	Age
	6	7	8	9	10	11	12
	*n* = 2	*n* = 15	*n* = 27	*n* = 36	*n* = 13	*n* = 10	*n* = 5
Eye number	2	15	27	36	13	10	5
Initial axial length (AL1) (mm)	23.11 ± 0.38	24.02 ± 0.40	24.12 ± 0.64	24.21 ± 0.67	24.35 ± 0.53	24.52 ± 0.48	23.93 ± 0.87
Axial length after 1 year (AL2) (mm)	23.37 ± 0.46	24.26 ± 0.43	24.25 ± 0.68	24.33 ± 0.71	24.42 ± 0.46	24.68 ± 0.54	23.99 ± 0.76
Axial length elongations (ΔAL) (mm)	0.26 ± 0.08	0.24 ± 0.17	0.13 ± 0.13	0.12 ± 0.16	0.07 ± 0.20	0.16 ± 0.11	0.06 ± 0.11
Initial Spherical equivalent refraction, SER1 (D)	−1.00 ± 0.35	−1.43 ± 0.71	−1.36 ± 0.60	−1.61 ± 0.82	−1.68 ± 1.04	−1.69 ± 1.01	−1.88 ± 0.75
Spherical equivalent refraction after 1 year, SER2 (D)	−1.25 ± 0.35	−1.71 ± 0.82	−1.53 ± 0.68	−1.78 ± 0.96	−1.73 ± 1.15	−2.04 ± 1.07	−1.95 ± 0.60
Spherical equivalent refraction progression (ΔSER) (D)	−0.25	−0.29 ± 0.34	−0.17 ± 0.28	−0.17 ± 0.44	−0.04 ± 0.52	−0.35 ± 0.23	−0.08 ± 0.42

**Table 3:** Baseline and 1‑year follow‑up data by age within the SVL group.

Variable	Age	Age	Age	Age	Age
	7	8	9	10	11
	*n* = 6	*n* = 9	*n* = 15	*n* = 9	*n* = 6
Eye number	6	9	15	9	6
Initial axial length (AL1) (mm)	23.75 ± 0.68	23.76 ± 1.17	24.48 ± 0.86	23.94 ± 0.44	24.28 ± 1.11
Axial length after 1 year (AL2) (mm)	24.14 ± 0.77	24.23 ± 1.25	24.75 ± 0.95	24.22 ± 0.49	24.51 ± 1.17
Axial length elongations (ΔAL) (mm)	0.39 ± 0.19	0.46 ± 0.19	0.28 ± 0.19	0.28 ± 0.11	0.23 ± 0.08
Initial spherical equivalent refraction, SER1 (D)	−1.17 ± 0.80	−1.07 ± 0.63	−1.43 ± 0.53	−1.47 ± 1.18	−1.60 ± 0.98
Spherical equivalent refraction after 1 year, SER2 (D)	−1.98 ± 0.70	−1.93 ± 0.77	−2.18 ± 0.78	−2.08 ± 1.34	−2.08 ± 1.15
Spherical equivalent refraction progression (ΔSER) (D)	−0.81 ± 0.47	−0.86 ± 0.42	−0.74 ± 0.44	−0.61 ± 0.49	−0.48 ± 0.34

### 3.2 Comparisons between the SVL and HAL groups

The SVL group was compared with the HAL group for age, sex, axial length (AL), and spherical equivalent (SE) at baseline (Table 1). AL was normally distributed but showed heterogeneity of variance, whereas SE displayed a skewed distribution; therefore, non-parametric tests (Mann–Whitney *U*) were applied. Mann–Whitney *U* tests were used to evaluate differences between the HAL group (*n* = 108) and the SV group (*n* = 49) on the six indicators. Baseline characteristics (sex and age) were well balanced, ensuring that observed differences were not confounded. For the changes during follow-up: ΔAL and ΔSE differed significantly between the two groups (*p* < 0.001) with medium effect sizes (*r* ≈ 0.37–0.39), both being markedly higher in the SVL group. Thus, for both axial length elongation and refractive progression, the HAL lenses outperformed the SVL lenses; the HAL group demonstrated significantly greater control of myopia progression.

### 3.3 Comparison within the HAL group

**Figure 1:**
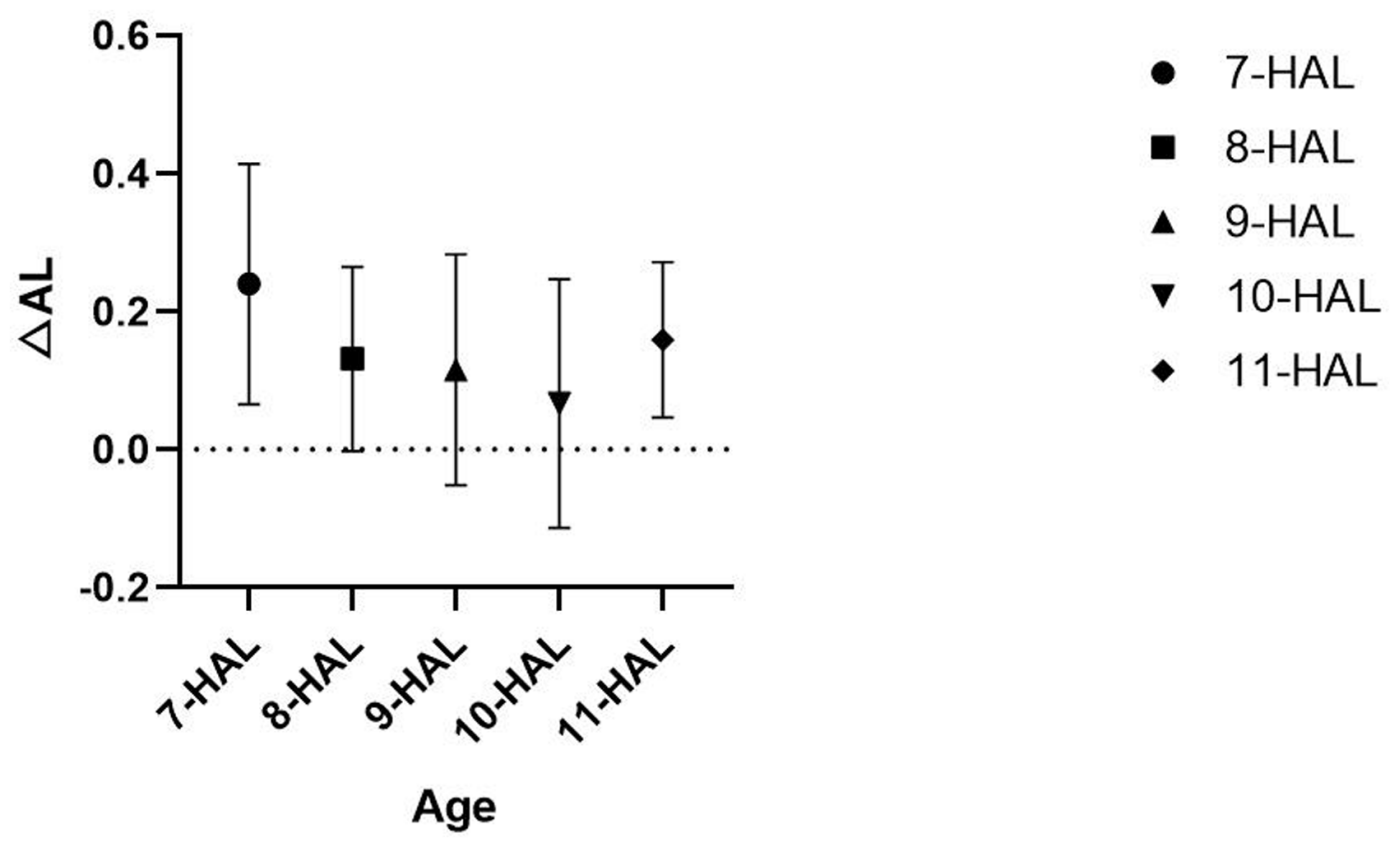
Within the HAL group, the mean ΔAL decreases with increasing age. ΔAL in the 7-year group was significantly higher than in the 8-year (*p* = 0.03), 9-year (*p* = 0.009), and 10-year (*p* = 0.003) groups.

Owing to the small numbers in the 6- and 12-year subgroups and differing baseline values among the 10-, 11-, and 12-year groups, statistical evaluation could not be performed. All 7–11 year groups were normally distributed; homogeneity of variance was confirmed for SE and ΔSE (*p* > 0.05) but not for AL and ΔAL. Consequently, non-parametric Kruskal–Wallis *H* tests were employed for single-factor comparisons. A significant inter-group difference was found for ΔAL (*p* = 0.003), whereas AL, SE, and ΔSE showed no age-related differences (p > 0.05). DSCF post-hoc tests indicated that the median ΔAL in the 7-year group was significantly higher than in the 8-year (*p* = 0.03), 9-year (*p* = 0.009), and 10-year (*p* = 0.003) groups; no difference was detected between the 8-year, 9-year and 10-year groups (*p* = 0.652, 0.190, 0.308). The effect size was *ε*^2^ = 0.055 (small-to-medium), suggesting that the age effect on ΔAL is largely confined to the 7-year period, with changes plateauing after 8 years of age (Table 4, Figure 1).

**Table 4:** Comparison of axial length elongation of different ages within the HAL group.

Age	7	8	9	10	11	
8	0.03					*p*-value
9	0.009	0.652				*p*-value
10	0.003	0.190	0.308			*p*-value
11	0.158	0.755	0.520	0.183		*p*-value
12	0.031	0.394	0.530	0.354	0.338	*p*-value

### 3.4 Comparison within the SVL group

**Figure 2:**
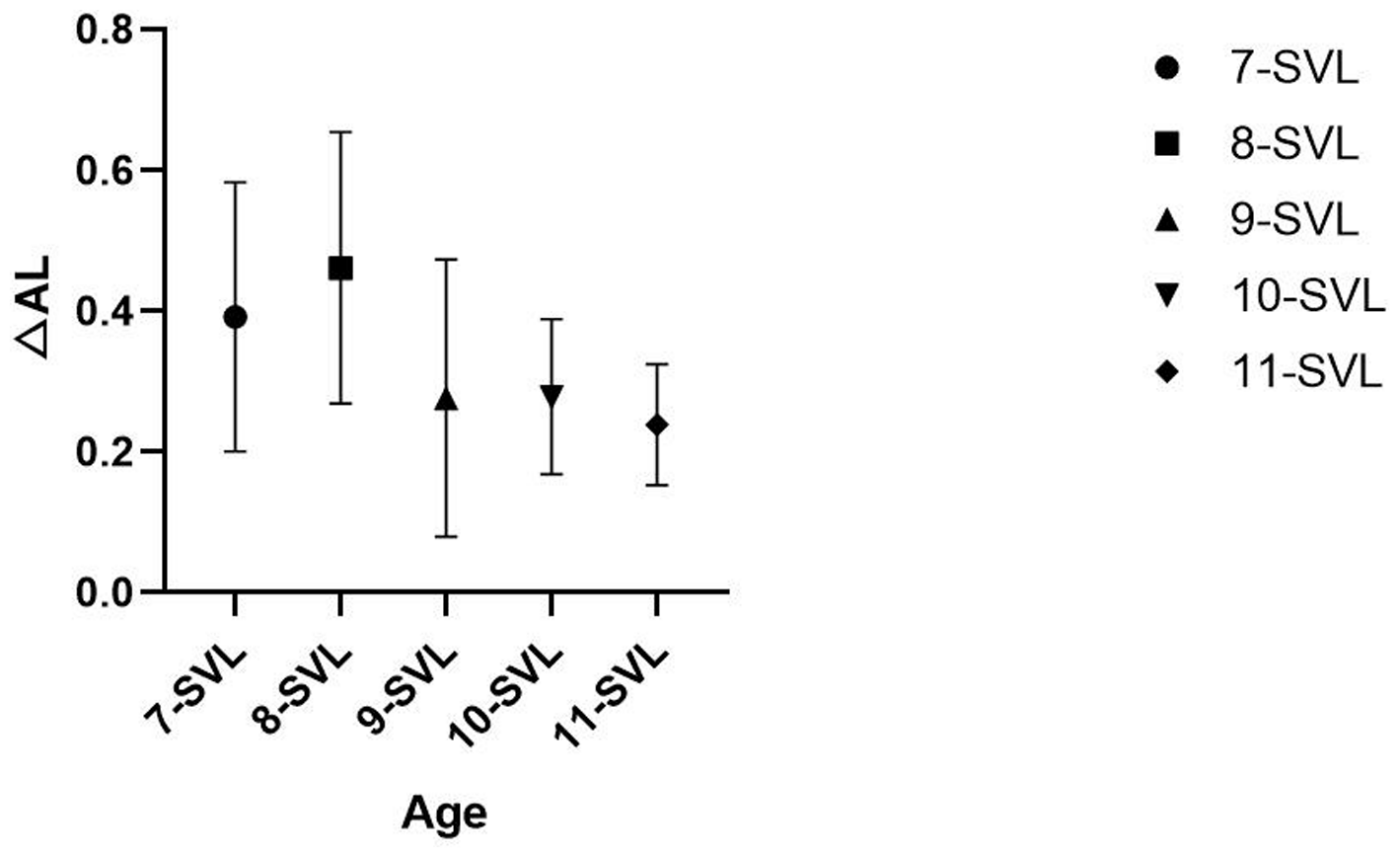
Within the SVL group, the mean ΔAL also decreases with increasing age. The 7- and 8-year-old group had significantly higher ΔAL scores than the 9-, 10-, and 11-year-old groups (*p* < 0.05), but there is no difference between 7 and 8-year old groups.

For the SVL group, a general linear model was used to analyze the effect of age on ΔAL while controlling for AL1. The results showed that the overall model explained a very high proportion of variance (adjusted *R*^2^ = 0.972, *R*^2^ = 0.972). The assumption of homogeneity of variance was met (Levene’s test: *F*(4,40) = 1.830, *p* = 0.142 *F*(4,40) = 1.830, *p* = 0.142). AL1 had a strong positive predictive effect on AL2 (*B* = 1.070, *p* < 0.001, *B* = 1.070, *p* < 0.001), and the main effect of age was also significant (*F*(4,39) = 3.863, *p* = 0.010 *F*(4,39) = 3.863, *p* = 0.010). Further pairwise comparisons (LSD) indicated that the 8-year-old group had significantly higher ΔAL scores than the 9-, 10-, and 11-year-old groups (*p* < 0.05), and the 7-year-old group had significantly higher ΔAL scores than the 9- and 11-year-old groups (*p* < 0.05), with no significant differences among the remaining age groups (*p* > 0.05) (Table 5, Figure 2).

**Table 5:** Comparison of axial length elongation of different ages within the SVL group.

Age	7	8	9	10	
8	0.423				*p*-value
9	0.044	0.002			*p*-value
10	0.142	0.014	0.507		*p*-value
11	0.049	0.005	0.764	0.462	*p*-value

## 4 Discussion

The myopia is irreversible; therefore, the control and prevention of myopia is an essential part of its management. Currently, there are three widely used and effective control measures, namely, the use of corneal contact lenses (multifocal soft contact lenses, orthokeratology lenses), spectacle lenses (HAL, DIMS and progressive lenses), and low-concentration atropine (7). In China, the most widely used methods are orthokeratology lenses, peripheral defocus lenses (including DIMS, HAL and Diffusion Optics Technology (DOT) lenses), and low-dose atropine. In this retrospective study, we primarily compared HAL lenses with SVL and investigated how age influences myopia control within the HAL group.

SVL have long been the standard correction, yet their ability to slow myopia progression is limited. Qin et al. (8) conducted a retrospective study of 773 children with myopia (aged, 6–10 years) in China who wore single vision lenses for 6–7 years and found that the average increase in myopic spherical equivalent was −0.60 ± 0.21 D per year, with a higher likelihood of developing high myopia in those who were younger and had higher myopia at the initial diagnosis. Zhang et al. (9) conducted a study in Hangzhou, China, and compared single vision lenses with a control group comprising 1,685 primary school students who took no measures, and concluded that SVL had no significant effect on the progression of myopia in children with low myopia. Collectively, these findings indicate that SVL provide only weak myopia control.

In our study, both SE and AL differed significantly between the SVL group and the HAL group, demonstrating a markedly superior myopia-control effect with the HAL lenses. Multiple randomized controlled trials have proven (10, 11) that HAL lenses are significantly more effective compared to SVL in terms of axial length elongation and spherical equivalent refraction progression. The findings of these studies are consistent with those of our retrospective study regarding the limitations of single vision lenses in myopia control and the effectiveness of HAL lenses. Although SVL are widely used in China due to their lower cost and long history, our findings suggest that for patients for whom it is financially feasible, HAL lenses and other myopia-control options such as DIMS and DOT lenses (12–15) should be the first-line choice to achieve better myopia-management outcomes. Specially designed lenses (HAL, DIMS, DOT) should be recommended first instead of single vision lenses to achieve better myopia control effects.

We further examined the role of age within the HAL and SVL groups. In the HAL group, 7-year-olds exhibited faster axial elongation than those aged 8, 9, or 10 years, whereas in the SVL group, 8-year-olds showed faster axial elongation than those aged 9, 10, or 11 years. These findings suggest that regardless of the type of lenses worn, younger age is associated with greater increases in both refractive error and axial length (Figures 1, 2). Age itself is a known factor influencing myopia progression; according to Noel et al. (16), each additional year of age is associated with a 15% reduction in axial elongation. Although the limited sample size precludes using any specific age as an absolute reference for all individuals, the data nonetheless highlight that even under the same intervention, myopia progression varies across age groups. Therefore, for younger children—particularly those under 8 years of age, combination strategies such as low-concentration atropine may need to be considered. Within the HAL group, axial elongation at age 11 showed an increase, while in the SVL group it continued to decrease. We speculate that this difference may be attributable to the limitations of the sample size.

In our study, both groups presented with low myopia at baseline (mean SE -1.38 ± 0.80 D and −1.54 ± 0.79D, respectively), and the HAL group demonstrated effective control in this low-myopia range (0.13 ± 0.16 mm). Previous reports have shown that HAL lenses yield better efficacy in low myopia (17). Similar findings have been described for DIMS lenses, where lower baseline myopia was associated with smaller axial length increases: Lam’s randomized controlled trial reported that within 1 year, axial elongation was 0.11 ± 0.02 mm and SER change was −0.17 ± 0.05 D, compared with 0.32 ± 0.02 mm and −0.55 ± 0.04 D, respectively, in the SVL group (13). As for the moderate to high myopia, Tang’s study (18) revealed that After 12 months, mean AL elongation was 0.21 ± 0.15 mm in the HAL group, and 0.28 ± 0.15 mm in the SVL group. Collectively, these observations suggest that, regardless of the specific intervention, lower initial myopia is associated with more favorable control outcomes.

In our study, changes in SE did not differ significantly across age subgroups (*p* > 0.05), in contrast to the clear differences observed for axial length. This discrepancy may be attributable to three factors: (1) the modest sample size, which should be enlarged in future work; (2) potential measurement variability in refraction, suggesting that a single, trained optometrist should perform all assessments hereafter; (3) the possibility that small increments in axial length do not immediately translate into measurable shifts in refractive error, especially when axial elongation remains within a clinically limited range.

## 5 Conclusion

Single-vision lenses provide only minimal control of myopia progression, whereas Specially designed lenses such as the HAL lenses effectively slow myopia development—especially by curbing axial elongation. Age is also a critical factor: even with HAL lenses, axial growth remains pronounced in children under 8 years and is more difficult to control. For those who develop myopia at a younger age, combined pharmacological interventions may offer a better strategy than optical correction alone.

## Data Availability

The raw data supporting the conclusions of this article will be made available by the authors, without undue reservation.
